# A Bibliometric Analysis of the Rise of ChatGPT in Medical Research

**DOI:** 10.3390/medsci11030061

**Published:** 2023-09-17

**Authors:** Nikki M. Barrington, Nithin Gupta, Basel Musmar, David Doyle, Nicholas Panico, Nikhil Godbole, Taylor Reardon, Randy S. D’Amico

**Affiliations:** 1 Chicago Medical School, Rosalind Franklin University, North Chicago, IL 60064, USA; 2 School of Osteopathic Medicine, Campbell University, Lillington, NC 27546, USA; 3 Faculty of Medicine and Health Sciences, An-Najah National University, Nablus P.O. Box 7, West Bank, Palestine; 4 Central Michigan College of Medicine, Mount Pleasant, MI 48858, USA; 5 Lake Erie College of Osteopathic Medicine, Erie, PA 16509, USA; 6 School of Medicine, Tulane University, New Orleans, LA 70112, USA; 7 Department of Neurology, Henry Ford Hospital, Detroit, MI 48202, USA; 8 Department of Neurosurgery, Lenox Hill Hospital, New York, NY 10075, USA

**Keywords:** ChatGPT, large language models, artificial intelligence, medicine, research

## Abstract

The rapid emergence of publicly accessible artificial intelligence platforms such as large language models (LLMs) has led to an equally rapid increase in articles exploring their potential benefits and risks. We performed a bibliometric analysis of ChatGPT literature in medicine and science to better understand publication trends and knowledge gaps. Following title, abstract, and keyword searches of PubMed, Embase, Scopus, and Web of Science databases for ChatGPT articles published in the medical field, articles were screened for inclusion and exclusion criteria. Data were extracted from included articles, with citation counts obtained from PubMed and journal metrics obtained from Clarivate Journal Citation Reports. After screening, 267 articles were included in the study, most of which were editorials or correspondence with an average of 7.5 +/− 18.4 citations per publication. Published articles on ChatGPT were authored largely in the United States, India, and China. The topics discussed included use and accuracy of ChatGPT in research, medical education, and patient counseling. Among non-surgical specialties, radiology published the most ChatGPT-related articles, while plastic surgery published the most articles among surgical specialties. The average citation number among the top 20 most-cited articles was 60.1 +/− 35.3. Among journals with the most ChatGPT-related publications, there were on average 10 +/− 3.7 publications. Our results suggest that managing the inevitable ethical and safety issues that arise with the implementation of LLMs will require further research exploring the capabilities and accuracy of ChatGPT, to generate policies guiding the adoption of artificial intelligence in medicine and science.

## 1. Introduction

Large Language Models (LLMs) are machine learning models designed to generate text that resembles human language. LLMs have attracted a great deal of interest in the medical field [1] as they have the potential to revolutionize medical research, patient care, and education by processing vast quantities of data faster and more precisely than humans can [1,2,3]. One of the most common LLMs is OpenAI’s ChatGPT (Chat Generative Pre-trained Transformer) [4] which has been increasingly investigated for its use in medical research [5], practice [3], and education [2]. As its name implies, ChatGPT employs transformer architecture, which was introduced in 2017 [6] as a means to overcome the limitations of previous Artificial Neural Network (ANN) models, such as Recurrent Neural Networks (RNNs) and Long Short-Term Memory Networks (LSTMs) [7], specifically their limitations with regard to understanding the context of a given language input (for example, understanding that the phrase “The bark was loud” refers to an animal noise, rather than the bark of a tree). Transformer architecture employs self-attention to contextualize words, matching one vector known as a query for a given word to a vector known as a key for another word, thereby indicating that the second word is relevant to the first in terms of context [6,8]. The weight of this relevance is indicated by a third vector known as the value, and helps to determine which keys are most relevant to the query (in our example above, these vectors would allow for understanding that “bark” in the context of “loud” refers to a noise, rather than an object) [6,8]. 

Since its introduction in November 2022, ChatGPT (version 3.5), armed with the capabilities of transformer architecture, has garnered a great deal of interest as a means to increase efficiency in medical practice and accelerate medical research. As an LLM, ChatGPT employs vast amounts of training data to “learn” and improve its predictive capabilities based on the context of the inputs provided, producing accurate and seemingly thoughtful responses to text prompts based on complex algorithms [9]. As such, it is intended to simulate human intelligence processes. However, despite its increasing ability to understand context, one of the biggest criticisms of the platform is that ChatGPT’s goal is not necessarily to produce correct answers to user queries, but rather to produce text that reads as though a human wrote it [9].

ChatGPT reached over 100 million users within two months of its launch [10] and was quickly trialed in both academic and medical settings [11,12], prompting a variety of ethical concerns around plagiarism [13], authorship [14,15], and dissemination of misinformation [16]. With its remarkable abilities, ChatGPT has already demonstrated success in passing specialized exams [17,18,19], including medical board exams [2], and has been credited with authorship on research articles [20], forcing educators and publishers to rapidly evolve teaching methods and publication policies to keep up with AI [21]. 

Despite these limitations, ChatGPT shows promise for use in healthcare settings, provided it is integrated mindfully. ChatGPT can be used as a search engine for both patients and healthcare professionals, for medical education, and for patient monitoring [14,22]. For healthcare professionals, ChatGPT could function as a powerful tool to remain up to date on the vast amounts of new scientific literature published daily, and to structure their critical analysis of such literature [23,24]. In medical education, ChatGPT could be similarly employed to structure preparation for standardized exams and summarize information for high-yield review [25,26]. For patients, ChatGPT may provide a tool to access health information on simple topics or conditions, or to clarify and summarize complex medical literature that would otherwise be challenging to comprehend [27,28]. Advancing ChatGPT’s transformer architecture by training it on additional parameters with each subsequent release (117 million for GPT-1, 1.5 billion for GPT-2, 175 billion for GPT-3) expands its ability to interpret the context of a string or block of text input and respond accordingly [29], allowing for increased precision in healthcare settings where accuracy is vital to patient safety and provider knowledge. 

The release of ChatGPT-4 in February 2023 further expanded ChatGPT’s capabilities, particularly with regard to its accuracy, increasing ChatGPT test scores on standardized exams and decreasing the frequency of fabricated information [29]. ChatGPT-4 also allows for inputs in the form of images or data rather than text only [29], expanding its ability to generate text based on a body of data, which certainly has implications for scientific research, particularly manuscript writing, and may also improve ChatGPT’s diagnostic capabilities in response to patient data input (demographics, exam findings, lab values, imaging, etc.).

Given the exponential increase in articles published on the use of ChatGPT in academia and medicine, this bibliometric analysis seeks to report on the current state of ChatGPT literature in these settings, evaluating reports of ChatGPT’s use in health care and research endeavors in various countries and specialties. Further, it will examine the advantages and pitfalls of ChatGPT in patient care, medical education, and scientific research. 

## 2. Materials and Methods

A title, abstract, and keyword search of PubMed, Embase, Scopus, and Web of Science databases was conducted using the search term “(“ChatGPT”) AND (med* OR surg* OR physician OR doctor OR patient)”. Criteria for inclusion were: (1) primary literature on ChatGPT in the medical field (including patient care, medical research, and medical education), (2) peer-reviewed, and (3) published in English. Articles were excluded based on the following exclusion criteria: (1) not primary literature on ChatGPT in the medical field, (2) primary literature on ChatGPT use in basic science and non-medically focused academic applications, (3) articles written by ChatGPT (and not about ChatGPT itself), (4) not peer reviewed (abstract, poster, published in preprint server, etc.), (5) not published in English. 

Articles were uploaded to Rayyan [30] and duplicates were removed. Title and abstract screening were conducted by two independent and blinded reviewers (NG and BM). Afterwards, conflicts were resolved by a third independent reviewer (NB). 

Included articles were subjected to a data extraction process. To ensure homogenous data, citation counts for all articles were obtained from PubMed and journal metrics were obtained from Clarivate Journal Citation Reports. Further variables collected included date of publication, journal, country of origin for first and last author, total citations, medical specialty/subspecialty, topic of article, study type, and journal metrics. For publication data, the month of official e-publication was taken (rather than online-ahead-of-print or early online preview dates). Journal metrics were obtained using the 2023 Journal Citation Report [31]. Data analysis and figure generation were completed using Microsoft Excel, Google Sheets, and Python. 

## 3. Results

### 3.1. Search Results

**Figure 1:**
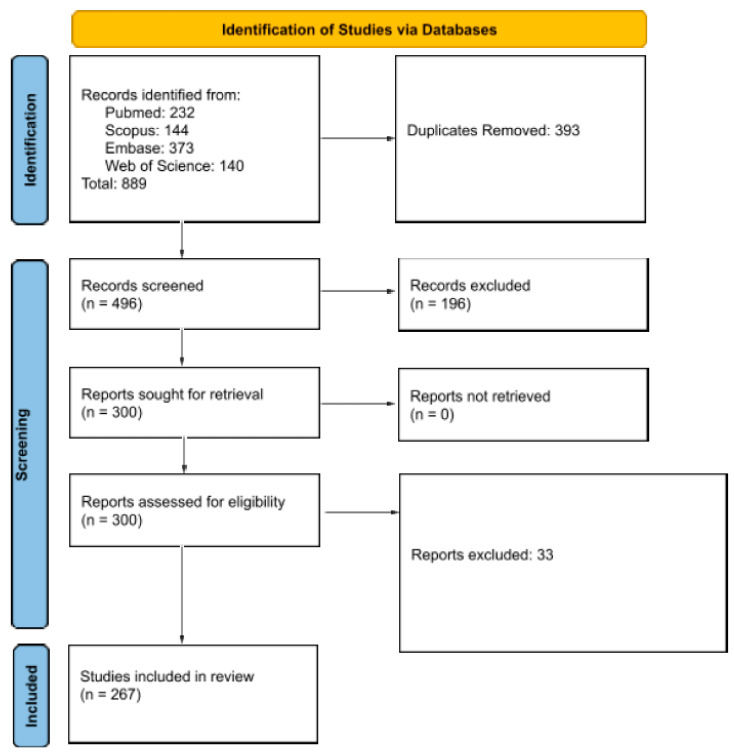
PRISMA diagram demonstrating screening strategy for included articles.

The initial search results yielded 889 total results and, after duplicates were removed, 393 articles remained for screening. Upon the initial title and abstract screen, 196 articles were excluded, followed by 33 exclusions upon full text review. Finally, 267 articles were included in the study (Figure 1). 

### 3.2. General Characteristics, Temporal and Geographic Trends 

The types of articles included were mainly letters to the editor/correspondence (*n* = 91, 35.2%) and commentary/editorials (*n* = 81, 31.3%). Articles, including observational and survey studies, comprised 78 articles (30.2%). Finally, case reports comprised six articles (2.3%). The included articles had a total of 1975 citations, ranging from 0 to 147 citations. The mean number of citations was 7.5 +/− 18.4 per publication, with a median of one citation. Finally, there were 177 unique journals represented. 

**Figure 2:**
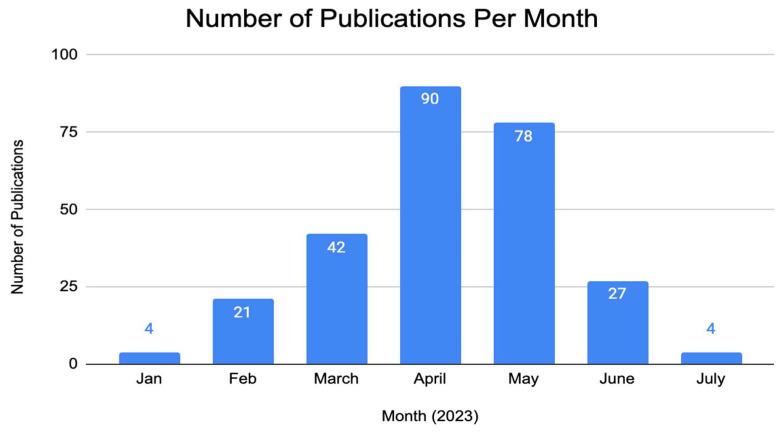
Number of publications on ChatGPT in the medical field per month since the beginning of 2023.

As the use of ChatGPT is relatively novel, all publications included in this study were from 2023. Therefore, temporal trends were evaluated by month, rather than year (as traditionally seen in bibliometric analyses) (Figure 2). The number of publications on ChatGPT in medicine steadily increased from January 2023 (4 publications) to March 2023 (42 publications), reaching a peak in April 2023 (90 articles). Our search indicated a lower number of articles published in May and June 2023 (78 and 27, respectively). 

**Figure 3:**
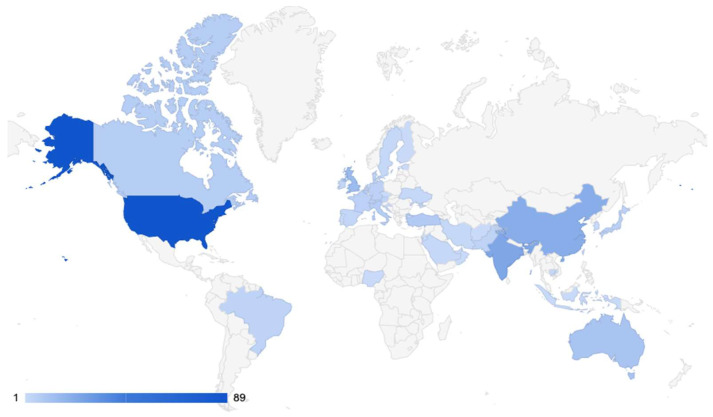
Geospatial map showing the location of senior authors publishing on ChatGPT in medicine.

For geospatial trends in publications on ChatGPT in medicine, the country of the senior author was recorded and is presented in Figure 3. The United States had the highest number of publications (89), followed by India (24), China (21), and the United Kingdom (15). 

### 3.3. Topics and Medical Specialties

**Figure 4:**
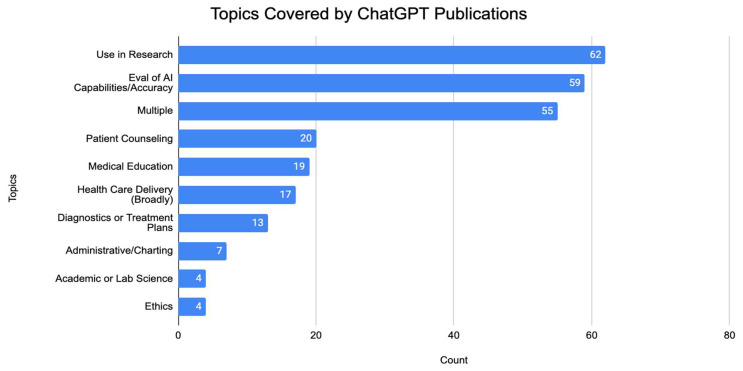
Topics of ChatGPT publications.

Articles were probed to determine the focus of the content, with most articles mainly concerned about the use of ChatGPT in research (62). The second most common topic was the evaluation of ChatGPT capabilities and accuracy (59). Examples of these included studies such as “Artificial Hallucinations in ChatGPT: Implications in Scientific Writing” and “Comparing Physician and Artificial Intelligence Chatbot Responses to Patient Questions Posted to a Public Social Media Forum”. Other common topics included seeking to understand the use of ChatGPT in patient counseling (20) and medical education (19). Examples of studies which looked at these topics included “Artificial intelligence chatbots will revolutionize how cancer patients access information: ChatGPT represents a paradigm-shift” and “How Does ChatGPT Perform on the United States Medical Licensing Examination? The Implications of Large Language Models for Medical Education and Knowledge Assessment”, respectively. There were 55 articles which fit multiple topics, with common combinations including the use in research and evaluation of the accuracy of ChatGPT, and patient counseling and diagnostic/treatment plans. These data are summarized in Figure 4.

For the application of ChatGPT, specifically to aid in certain medical fields, there were 20 non-surgical and 12 surgical specialties represented (Table 1). For non-surgical specialties, the application of ChatGPT was most published in radiology (including both diagnostic and interventional) (21, 25.3%). The second most common was internal medicine/primary care (10, 12.0%), followed by oncology (6, 7.2%). For surgical applications/specialties, plastic surgery had the highest number of articles (18, 26.9%). General surgery had the second most (15, 22.4%), followed by orthopedic surgery and ophthalmology, (7, 10.4% and 6, 9.0%, respectively). For neurosurgery specifically, there were four articles (6.0%).

### 3.4. Citation Metrics and Characteristics of Top 20 Cited Studies 

The top 20 most-cited articles for the use of ChatGPT in medicine ranged in citation count from 147 (“How Does ChatGPT Perform on the United States Medical Licensing Examination? The Implications of Large Language Models for Medical Education and Knowledge Assessment”) to 25 citations (“Assessing the performance of ChatGPT in answering questions regarding cirrhosis and hepatocellular carcinoma”) (Table 2). The average number of citations was 60.1 +/− 35.3 and the majority (14) of articles were published during February and March of 2023. Geographic analysis of the top articles revealed substantial contributions from countries such as the United States and United Kingdom, with six and four articles, respectively. Countries with a more minor contribution included European countries (such as Italy, Belgium, and France), Australia, Pakistan, and Taiwan. Finally, the most common type of article in the top 20 most cited were editorials and letters to the editor.

The most common topic within the top cited articles was the use of ChatGPT in research (9 of 20). Other topics which were covered included the evaluation of ChatGPT’s capabilities, diagnosis and treatment plans, and the use of ChatGPT for reducing the burden of administrative tasks for physicians. As the most common topic was the application of ChatGPT to research in general, 12 of the 20 studies were not specific to a singular medical specialty. Among the papers that investigated the application of ChatGPT to a specific specialty, the most common was surgery (two general surgery, one orthopedic surgery, and one neurosurgery). Interestingly, both the neurosurgical and orthopedic surgery articles focused mainly on research, a large aspect of both specialties. The three other medical specialties represented in the top 20 included internal medicine, hepatology, and infectious disease. 

### 3.5. Top Journals and Keywords 

The top five journals with the highest number of related publications contained on average 10 +/− 3.7 publications, with an average impact factor and h-index of 150.8 +/− 117.6 and 8.6 +/− 8.3, respectively (Table 3). The journal with the highest number of articles was The Annals of Biomedical Engineering, containing 14 articles (IF = 3.8). Following this was the Aesthetic Surgery Journal (13 articles, IF = 2.9), and Cureus (10 articles, IF = 1.15). 

Within the top 20 most-cited articles, the most common journal was The Lancet, with three articles published in The Lancet Digital Health and one article published in The Lancet Infectious Disease. The top five journals with the highest number of related publications contained on average 10 +/− 3.7 publications, with an average h-index and impact factor of 150.8 +/− 117.6 and 8.6 +/− 8.3, respectively (Table 3). The journal with the highest number of articles was The Annals of Biomedical Engineering, containing 14 articles (h-index = 141, IF = 3.8). Following this was the Aesthetic Surgery Journal (13 articles, h-index = 71, IF = 2.9), and Cureus (10 articles, h-index = NA, IF = 1.15). Other journals with significant contributions included Radiology (8 articles, h-index = 320, IF = 19.7) and the International Journal of Surgery (5 articles, h-index = 71, IF = 15.3).

**Figure 5:**
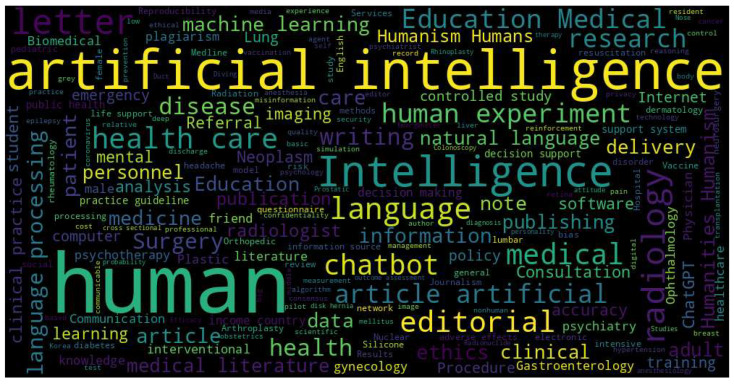
Visual map of the top keywords used in included studies. Larger font indicates higher frequency of keyword usage.

Finally, to visually represent the top keywords which were used for articles included in this study, a keyword cloud map was generated (Figure 5). 

## 4. Discussion

The abrupt emergence of LLMs into public awareness and their rapid adoption in academics and medicine has highlighted their applicability and compelled careful evaluation of the ethical concerns raised by this powerful technology. Although there are a multitude of LLMs which are emerging, OpenAI’s ChatGPT is among the most popular, surpassing 100 million users within two months of release [32]. As such, its use among the medical research community and patients has led to the production of large amounts of literature. Therefore, we performed a comprehensive bibliometric analysis of ChatGPT to better understand the response of the scientific and medical community to the use of LLMs for both academic and clinical pursuits. 

The increasing use of LLMs in academic and medical settings is highlighted by the types of publications appearing in our analysis, with editorials and correspondence publications identified as the most common article types. This is likely the result of an urgent need for dialogue on the implications of these unique rapidly materializing technologies, that in the case of ChatGPT became the fastest-growing consumer application in mere months [10]. This dialogue was necessary to initiate discussions on the potential benefits and risks of incorporating LLMs, and the need to build a foundation within medicine for its use. These commentaries initially outpaced the ability to perform observational and controlled studies evaluating the technology’s capabilities and accuracy. The editorials and correspondences published have often emphasized concerns regarding the use of ChatGPT in academics and medicine, particularly surrounding authorship, plagiarism, and patient safety issues such as the spread of misinformation. Given these concerns, it is unsurprising that the most-cited articles were those that put ChatGPT to the test using medical board exams or questions about specific medical conditions. As pointed out in many of the editorial publications included in this study, there is a paucity of information about ChatGPT’s accuracy regarding medical topics, and the few studies that tested ChatGPT’s capabilities in a controlled way to better understand its limitations were cited by many other articles. As such, the immense potential of ChatGPT must be carefully tempered to safeguard against misinformation and potential biases generated by the datasets used to train ChatGPT.

In terms of geography and medical specialties, the United States, India, and China had the highest number of ChatGPT-related publications, while European countries had lower numbers of articles. This is likely representative of the significant research output of these countries in general and may also be reflective of the prevalence of artificial intelligence research in the U.S. and China in particular [33,34,35]. Interestingly, radiology was the most represented specialty among ChatGPT-related articles, which may suggest an interest in exploring whether artificial intelligence could be used to accelerate the creation of radiology reports, enabling clinicians to redistribute their time toward assessing images [36,37]. Second to radiology was internal medicine, a field in which artificial intelligence could be used similarly to expedite clinical charting [38], generate differential diagnoses, or provide patients with basic information about their diagnoses, provided the information is accurate [39,40]. Given the hands-on and highly technical nature of surgical specialties, it is interesting, although perhaps not surprising, that most articles emerging from these fields focused on using ChatGPT in research settings. In plastic surgery for example, ChatGPT may not be particularly useful in the operating room outside of generating operative notes, but it can accelerate academic productivity in fields that are highly competitive and value publication output. According to the 2022 NRMP Match Data [41], the highest number of publications were produced by applicants matching in plastic surgery and neurological surgery. For osteopathic medical students, neurological surgery had the highest number of publications per matched applicant. Our data reflect the large number of plastic surgery publications; however, it is interesting to note the lack of neurosurgical publications—even with the “publication arms race” for medical students applying for neurosurgery [42]. 

Though the potential of ChatGPT is vast, our analysis makes clear the need for observational controlled studies to fully evaluate the capabilities and accuracy of ChatGPT in academic, medical, and research settings. Many of the editorials included in our analysis discussed the importance of purposeful and ethical integration of artificial intelligence in these settings to avoid compromising intellectual integrity. In competitive fields such as plastic surgery and neurosurgery, ChatGPT’s capabilities may add fuel to the “publication race” by encouraging the use of artificial intelligence to increase publication output, as it may be impossible for authors who choose not to use artificial intelligence to keep up with those who do [43]. While this is likely to occur more prominently among students and trainees seeking to match into residency or build their academic careers, as ChatGPT’s capabilities expand this will certainly impact the upper echelons of academic medicine and science, where eligibility for academic promotions or funding for scientific endeavors is often directly tied to publication output and grant writing productivity, both of which can be accelerated via artificial intelligence.

Ethical integration of ChatGPT into medicine and scientific research was among the most discussed topics in our study. These ethical considerations will not only require significant further study of ChatGPT itself, but will also require institutions, journals, and medical associations to formulate clear guidelines for the use of ChatGPT in each setting [20,44,45,46]. ChatGPT has incredible potential to reduce administrative burdens in medicine by assisting with charting and record-keeping [38], potentially enabling providers to focus more on the clinician-patient relationship that is often challenging to prioritize in modern medicine. However, it is essential to ensure that recorded information is accurate, and that diagnoses are not missed due to an over-reliance on artificial intelligence. Similarly, ChatGPT has the potential to curb the impact of our present healthcare worker shortage [47], specifically by providing patients with basic information that they might otherwise seek in primary care settings. In the age of misinformation, using ChatGPT in patient-facing formats must be evaluated and integrated with caution to ensure patients are given accurate information about their health [32], and appropriately directed to human providers when needed.

In research settings, transparency is vital to ensuring intellectual integrity, which may take the form of statements specifying where and how artificial intelligence was used within a publication, or stated limitations on the appropriate use of artificial intelligence when submitting a manuscript for publication. The scientific community will need to grapple with difficult questions when determining what is acceptable: where do we draw the line between ChatGPT writing assistance (outlining, syntax suggestions) and articles written entirely by ChatGPT? Is ChatGPT use allowable in training settings (from primary school through to graduate school and beyond) where the writing process itself is intended to be a learning experience? None of these questions have an easy answer, but as ChatGPT and other LLM platforms become ubiquitous, it may become necessary to redefine our conceptions of plagiarism, originality, and innovation to make room for both the enormous potential and dangerous pitfalls of artificial intelligence.

### Limitations

A recognized constraint in the methodology of bibliometric analysis is the necessity to rely on citations as a metric for analysis, as various factors influence the likelihood of being cited, such as the journal’s impact factor, the reputation of the authors involved, and the recency of publication [48]. Specifically, within the context of a novel technology such as ChatGPT, it is believed that the recentness of its adaptation has impacted the ability of relevant publications to be recognized, resulting in some potent articles likely being overlooked. Collectively, this could potentially lead to a lack of citations within these meaningful works, falsely deflating their relevance. 

Another limitation of our study is that we focused primarily on ChatGPT, which is currently the most prevalent LLM in the medical field. Other LLMs, such as Meta’s large language modeled meta-AI (LLaMA), have not been extensively reported on due to relatively recent deployment or less adoption by the medical community. For non-specialized LLMs such as LLaMA there are a small but growing number of reports in the literature which have opted to use it over ChatGPT because it is open source [49]. There are other, more specialized LLMs, such as the recently reported HealthSearchQA [50], Stanford’s BioMedLM [51], and RadBERT which is specifically for radiology [52], that could have significant impacts on the medical research community as well. Similarly, neurosurgical journals are utilizing novel and innovative approaches to artificial intelligence, by employing specialized conversational LLMs to aid readers in understanding manuscripts [53]. Given the novelty of these emerging platforms, these specialized LLMs were not evaluated in our study, as there are not enough studies to warrant an accurate bibliometric analysis. The extensive range of applications for large language models such as ChatGPT warrant extensive validation of these technologies across diverse settings in medicine and science, while concurrently establishing universal guidelines and ethical models for their efficient and responsible utilization.

## 5. Conclusions

As ChatGPT has had an explosive impact on the field of medicine, this study sought to evaluate the exponential increase in publications since the release of ChatGPT and its impact on the medical field. Our results suggest that there is a large focus on the application of ChatGPT in medical research, the accuracy and capabilities of ChatGPT, and its use in patient education/counseling. It is critical that future studies seek to thoroughly evaluate the impact and potential uses and misuses of ChatGPT, including ethical and legal implications. While current research has sought to exhibit the wide range of capabilities ChatGPT possesses, our review establishes that gaps exist in the literature with regards to demonstrating its specific limitations. Potential applications should also be further established in a specialty-specific context, as wide variations in research activity among specialties reveal significant potential for an exploration of ChatGPT as a patient care tool within many nonsurgical specialties. Together, these developments in research will likely further establish and optimize the niche of LLM in medicine. Our analysis suggests LLM accuracy and precision in healthcare and research settings will be the primary topic of discussion in this body of literature moving forward, and, given the rapid rollout of updated versions of ChatGPT and its peer LLM platforms, it will be essential for medical professionals and scientists to remain vigilant in ensuring the safe and ethical implementation of these technologies in an ever-changing healthcare landscape.

## Figures and Tables

**Table 1:** Number of publications produced by surgical and non-surgical specialties.

Medical Specialty	Number of Publications
**Non-Surgical**	**N (%)**
Radiology	21 (25.3%)
Internal Medicine/Primary Care	10 (12.0%)
Oncology	6 (7.2%)
Gastroenterology	5 (6.02%)
Rheumatology	5 (6.02%)
Dermatology	4 (4.8%)
Emergency Medicine	4 (4.8%)
Endocrine	4 (4.8%)
Pediatrics	4 (4.8%)
Psychiatry	4 (4.8%)
Neurology	3 (3.6%)
Anesthesia	2 (2.4%)
Infectious Disease	2 (2.4%)
Pathology	2 (2.4%)
Pulmonology/Critical Care	2 (2.4%)
Cardio	1 (1.2%)
Fam Med	1 (1.2%)
Hepatology	1 (1.2%)
Sports Med	1 (1.2%)
Toxicology	1 (1.2%)
**Surgical Specialties**	**N (%)**
Plastic Surgery	18 (26.9%)
General Surgery	15 (22.4%)
Orthopedic Surgery	7 (10.4%)
Ophthalmology	6 (9.0%)
Obstetrics and Gynecology	5 (7.5%)
Neurosurgery	4 (6.0%)
Otolaryngology	4 (6.0%)
Oral and Maxillofacial Surgery	2 (3.0%)
Surgical Oncology	2 (3.0%)
Urology	2 (3.0%)
Bariatric Surgery	1 (1.5%)
Colorectal Surgery	1

**Table 2:** Top 20 most-cited publications regarding the use of ChatGPT in medicine.

Rank	Article Name	Number of Citations	Journal of Article
1	How Does ChatGPT Perform on the United States Medical Licensing Examination? The Implications of Large Language Models for Medical Education and Knowledge Assessment.	147	*JMIR Medical Education*
2	A Conversation on Artificial Intelligence, Chatbots, and Plagiarism in Higher Education	119	*Cellular and Molecular Bioengineering*
3	Artificial Hallucinations in ChatGPT: Implications in Scientific Writing	118	*Cureus Journal of Medical Science*
4	ChatGPT: the future of discharge summaries?	94	*The Lancet Digital Health*
5	Can artificial intelligence help for scientific writing?	87	*Critical Care*
6	Evaluating the Feasibility of ChatGPT in Healthcare: An Analysis of Multiple Clinical and Research Scenarios.	68	*Journal of Medical Systems*
7	Role of Chat GPT in Public Health	61	*Annals of Biomedical Engineering*
8	ChatGPT: evolution or revolution?	57	*Medicine, Health Care, and Philosophy*
9	Generating scholarly content with ChatGPT: ethical challenges for medical publishing	56	*The Lancet Digital Health*
10	ChatGPT—Reshaping medical education and clinical management.	51	*Pakistan Journal of Medical Sciences*
11	The future of medical education and research: Is ChatGPT a blessing or blight in disguise?	48	*Medical Education Online*
12	Can ChatGPT draft a research article? An example of population-level vaccine effectiveness analysis.	45	*Journal of Global Health*
13	Comparing Physician and Artificial Intelligence Chatbot Responses to Patient Questions Posted to a Public Social Media Forum.	41	*JAMA Internal Medicine*
14	ChatGPT and other artificial intelligence applications speed up scientific writing.	37	*Journal of the Chinese Medical Association*
15	Revolutionizing radiology with GPT-based models: Current applications, future possibilities and limitations of ChatGPT.	32	*Diagnostic and Interventional Imaging*
16	Using ChatGPT to write patient clinic letters.	32	*The Lancet Digital Health*
17	Artificial intelligence bot ChatGPT in medical research: the potential game changer as a double-edged sword.	29	*Knee Surgery, Sports Traumatology, Arthroscopy*
18	ChatGPT and antimicrobial advice: the end of the consulting infection doctor?	28	*The Lancet Infectious Diseases*
19	To ChatGPT or not to ChatGPT? The Impact of Artificial Intelligence on Academic Publishing	26	*The Pediatric Infectious Disease Journal*
20	Assessing the performance of ChatGPT in answering questions regarding cirrhosis and hepatocellular carcinoma.	25	*Clinical and Molecular Hepatology*

**Table 3:** Characteristics of journals with the top 5 highest numbers of related publications.

Journal Name	Number of Related Publications	h-Index	Impact Factor
*Annals of Biomedical Engineering*	14	141	3.8
*Aesthetic Surgery Journal*	13	71	2.9
*Cureus*	10	NA	1.15
*Radiology*	8	320	19.7
*International Journal of Surgery*	5	71	15.3
Average +/− SD	10 +/− 3.7	150.8 +/− 117.6	8.6 +/− 8.3

## Data Availability

All data generated during the study are contained in this manuscript.
